# Fuzzy Adaptive Interacting Multiple Model Nonlinear Filter for Integrated Navigation Sensor Fusion

**DOI:** 10.3390/s110202090

**Published:** 2011-02-11

**Authors:** Chien-Hao Tseng, Chih-Wen Chang, Dah-Jing Jwo

**Affiliations:** 1 National Applied Research Laboratories, National Center for High-Performance Computing, 22 Keyuan Rd., Central Taiwan Science Park, Taichung City 40763, Taiwan; E-Mails: c00how00@nchc.org.tw (C.-H.T.); d9351002@nchc.org.tw (C.-W.C.); 2 Department of Communications, Navigation and Control Engineering, National Taiwan Ocean University, 2 Pei-Ning Rd., Keelung 202-24, Taiwan

**Keywords:** integrated navigation, unscented Kalman filter, interacting multiple model, fuzzy logic

## Abstract

In this paper, the application of the fuzzy interacting multiple model unscented Kalman filter (FUZZY-IMMUKF) approach to integrated navigation processing for the maneuvering vehicle is presented. The unscented Kalman filter (UKF) employs a set of sigma points through deterministic sampling, such that a linearization process is not necessary, and therefore the errors caused by linearization as in the traditional extended Kalman filter (EKF) can be avoided. The nonlinear filters naturally suffer, to some extent, the same problem as the EKF for which the uncertainty of the process noise and measurement noise will degrade the performance. As a structural adaptation (model switching) mechanism, the interacting multiple model (IMM), which describes a set of switching models, can be utilized for determining the adequate value of process noise covariance. The fuzzy logic adaptive system (FLAS) is employed to determine the lower and upper bounds of the system noise through the fuzzy inference system (FIS). The resulting sensor fusion strategy can efficiently deal with the nonlinear problem for the vehicle navigation. The proposed FUZZY-IMMUKF algorithm shows remarkable improvement in the navigation estimation accuracy as compared to the relatively conventional approaches such as the UKF and IMMUKF.

## 1. Introduction

A navigation filter is commonly designed by use of an extended Kalman filter (EKF) [1–3] to estimate the vehicle state variables and suppress the navigation measurement noise. Although it has been shown to be a minimum mean square error estimator, the fact that EKF highly depends on a predefined dynamics model forms a major drawback. For achieving good filtering results, the designers are required to have the complete *a priori* knowledge on both the dynamic process and measurement models, in addition to the assumption that both the process and measurement are corrupted by zero-mean Gaussian white sequences.

As a deterministic sampling approach, the unscented Kalman filter (UKF) [4–9] was first proposed by Julier, *et al*. [4] to address nonlinear state estimation in the context of control theory. The UKF is a nonlinear, distribution approximation method, which uses a finite number of carefully chosen sigma points to propagate the probability of state distribution through the nonlinear dynamics of system so as to completely capture the true mean and covariance of the Gaussian random variable (GRV) with a minimal set of samples. The UKF made a Gaussian approximation with a limited number sigma points by using the Unscented Transform (UT). The basic premise behind the UKF is it is easier to approximate a Gaussian distribution than it is to approximate an arbitrary nonlinear function. When the sample points are propagated through the true nonlinear system, the posterior mean and covariance can be captured accurately to the second order of Taylor series expansion for any nonlinear system. One of the remarkable merits is that the overall computational complexity of the UKF is the same as that of the EKF [8].

A high gain (high bandwidth) filter is needed to respond fast enough to the platform maneuvers while a low gain filter is necessary to reduce the estimation errors during the uniform motion periods. Under various circumstances where there are uncertainties in the system model and noise description, and the assumptions on the statistics of disturbances are violated due to the fact that in a number of practical situations, the availability of a precisely known model is unrealistic. One way to take them into account is to consider a nominal model affected by uncertainty. An a parametric adaptation approach, the adaptive Kalman filter (AKF) algorithm has been one of the strategies considered for estimating the state vector to prevent divergence problem due to modeling errors [9–11]. Many efforts have been made to improve the estimation of the covariance matrices based on the innovation-based estimation approach, resulting in the innovation adaptive estimation (IAE) [2,10,11]. Two popular types of the adaptive Kalman filter algorithms include the innovation-based adaptive estimation (IAE) approach [10,11] and the adaptive fading memory filter approach, which is a type of covariance scaling method. One of the adaptive fading memory filters is called the strong tracking Kalman filter [9], where the strong tracking algorithm (STA) involves a nonlinear smoother algorithm that employs suboptimal multiple fading factors.

The other major approach that has been proposed for AKF is the multiple model adaptive estimate (MMAE). An a structural adaptation approach, the interacting multiple model (IMM) algorithm [3,12,13] has the configuration that runs in parallel several model-matched state estimation filters, which exchange information (interact) at each sampling time. The IMM approach is based on filter structural adaptation (model switching). Based on a soft-switching framework, the IMM algorithm allows the possibility of using highly dynamic models just when required, diminishing unrealistic noise considerations in non-maneuvering situations of the system. The use of an IMM allows exploiting the benefits of high dynamic models in the problem of vehicle navigation. The IMM estimator obtains its estimate as a weighted sum of the individual estimates from a number of parallel filters matched to different motion modes of the platform. The objective is to design the nonlinear filter in an IMM algorithm suitable for high dynamic or curvilinear motions to navigate a maneuvering vehicle. Selected results presented in this paper confirm the improvements.

The IMM algorithm has been originally applied to target tracking [14–17], and recently extended to Global Positioning System (GPS) navigation [18,19], and integrated navigation designs [20–23]. A model probability evaluator calculates the current probability of the vehicle being in each of the possible modes. A global estimate of the vehicle’s state is computed using the latest mode probabilities. This algorithm carries out a soft-switching between the various modes by adjusting the probabilities of each mode, which are used as weightings in the combined global state estimate. The covariance matrix associated with this combined estimate takes into account the covariances of the mode-conditioned estimates as well as the differences between these estimates.

The UKF naturally suffers the same problem as the EKF. The uncertainty of the process noise and measurement noise will degrade the performance of the UKF. An adaptive mechanism which dynamically identifies uncertainties or modeling errors can be adopted. To deal with the noise uncertainty and system nonlinearity simultaneously, the IMMUKF can be introduced [24,25]. In the approach, these multiple models are developed to describe various dynamic behaviors. In each model an UKF is running, and the IMM algorithm makes uses of model probabilities to weight the inputs and output of a bank of parallel filters at each time instant. The fuzzy logic reasoning system is based on the Takagi-Sugeno (T-S) model. The fuzzy reasoning system is constructed for obtaining the suitable process noise according to the time-varying change in dynamics. By monitoring the innovation information, the Fuzzy logic adaptive system (FLAS) is employed for dynamically on-line determining better lower and upper bounds of the process noise covariance according to the innovation information, and therefore improves the estimation performance.

The synergy of Global Positioning System (GPS) and inertial navigation system (INS) has been widely explored due to their complementary operational characteristics [1,26]. The GPS/INS integrated navigation system, typically carried out through the EKF and UKF, is the adequate solution to provide a navigation system that has the superior performance in comparison with either the GPS or INS stand-alone system. The example on the tightly-coupled GPS/INS integrated navigation processing based on the FUZZY-IMMUKF will be presented. The performance comparison will be demonstrated by using the proposed FUZZY-IMMUKF method as compared to the relatively conventional UKF and IMMUKF approaches.

The remainder of this paper is organized as follows. In Section 2, the preliminary background on the interacting multiple model unscented Kalman filter for the navigation processing is discussed. The proposed sensor fusion strategy is introduced in Section 3. In Section 4, the navigation integration processing and performance evaluation are carried out to evaluate the performance comparison will be demonstrated using the proposed FUZZY-IMMUKF method as compared to the relatively conventional UKF and IMMUKF approaches. Conclusions are given in Section 5.

## 2. The Interacting Multiple Model Unscented Kalman Filter

The unscented Kalman filtering deals with the case governed by the nonlinear stochastic difference equations:

(1a) $$x_{k+1}=f(x_{k},k)+w_{k}$$

(1b) $$z_{k}=h(x_{k},k)+v_{k}$$

where the state vector **x***_k_* ∈ ℜ*^n^*, process noise vector **w***_k_* ∈ ℜ*^n^*, measurement vector **z***_k_* ∈ ℜ*^m^*, and measurement noise vector **v***_k_* ∈ ℜ*^m^*. The vectors **w***_k_* and **v***_k_* are zero mean Gaussian white sequences having zero cross-correlation with each other:

$$E[w_{k}\;w_{i}^{T}]=Q_{k}\;\delta_{\mathrm{ik}}\;;\;\;E[v_{k}\;v_{i}^{T}]=R_{k}\;\delta_{\mathrm{ik}}\;;\;\;E[w_{k}\;v_{i}^{T}]=0\;\;\;\;\;\;\;\;\;\;\mathrm{for all i and k}$$

where *E*[·] represents expectation, and superscript “T” denotes matrix transpose, **Q***_k_* is the process noise covariance matrix and **R***_k_* is the measurement noise covariance matrix. The symbol *δ_ik_* stands for the Kronecker delta function:

$$\delta_{\mathrm{ik}}=\{\begin{array}{ll} 1, & i=k\\ 0, & i\neq k \end{array}$$

The IMM approach takes into account a set of models to represent the system behaviour patterns or system model. The estimator carries out a ‘soft switching’ among various models by the model probability. The overall estimates is obtained by a combination of the estimates from the filters running in parallel based on the individual models that match the system modes. The measurements could be obtained from one or more sensors, and the model-matched filters could be linear or nonlinear. The algorithm of IMM-nonlinear filters is introduced to deal with the noise uncertainty and system nonlinearity simultaneously.

Let a general system for multiple models in discrete time be described by:

(2a) $$x_{k+1}=f(x_{k},k,M_{k})+w(x_{k},M_{k})$$

(2b) $$z_{k}=h(x_{k},k,M_{k})+v_{k}(x_{k},M_{k})$$

where *f*(·) and *g*(·) are the parameterized state transition and measurement functions, **x***_k_* and **z***_k_* are the dynamical state and measurement of the system in model *M_k_*, and the system itself is a Markov chain, **w**, **v** are the process noise and measurement noise with covariances **Q***_k_* and **R***_k_*, respectively.

Instead of linearizing using the Jacobian matrices as in the EKF and achieving first-order accuracy, the UKF uses a deterministic sampling approach to capture the mean and covariance estimates with a minimal set of sample points. The UKF was proposed to address nonlinear state estimation in the context of control theory. When the sample points are propagated through the true nonlinear system, the posterior mean and covariance can be captured accurately to the 3rd order of Taylor series expansion for any nonlinear system. One of the remarkable merits is that the overall computational complexity of the UKF is the same as that of the EKF [8].

The first step in the UKF is to sample the prior state distribution, *i.e.*, generate the sigma points through the UT, which is a method for calculating the statistics of a random variable which undergoes a nonlinear transformation. The basic premise is that to approximate a probability distribution is easier than to approximate an arbitrary nonlinear transformation. The samples are propagated through true nonlinear equations, and the linearization of the model is not required.

Suppose the mean **x̄** and covariance **P** of vector **x** are known, a set of deterministic vector called sigma points can then be found. The ensemble mean and covariance of the sigma points are equal to **x̄** and **P**. The nonlinear function **y** = *f* (**x**) is applied to each deterministic vector to obtain transformed vectors. The ensemble mean and covariance of the transformed vectors will give a good estimate of the true mean and covariance of **y**, which is the key to the unscented transformation. The UKF approach estimates are expected to be closer to the true values than the EKF approach.

The sigma vectors are propagated through the nonlinear function to yield a set of transformed sigma points:

(3) $$y_{i}=f(X_{i})\;i=0,...,2n$$

The mean and covariance of **y***_i_* are approximated by a weighted average mean and covariance of the transformed sigma points as follows:

(4) $${\bar{y}}_{u}=\sum_{i=0}^{2n}W_{i}^{\left(m\right)}y_{i}$$

(5) $${\bar{P}}_{u}=\sum_{i=0}^{2n}W_{i}^{\left(c\right)}(y_{i}-{\bar{y}}_{u}){\left(y_{i}-{\bar{y}}_{u}\right)}^{T}$$

As compared to the EKF’s linear approximation, the unscented transformation is accurate to the second order for any nonlinear function. The UKF algorithm is summarized in Appendix. Incorporation of the STA (also provided in Appendix) into the UKF results in the strong tracking unscented Kalman filter (STUKF).

The IMMUKF algorithm uses model (Markov chain state) probabilities to weight the input and output of a bank of parallel UKFs at each time instant. The approach takes into account a set of models to represent the system behavior patterns or system model. The overall estimates is obtained by a combination of the estimates from the filters running in parallel based on the individual models that match the system modes. An IMM cycle consists of four major stages: interaction (mixing), filtering, model probability calculation, and estimate combination, as described in the following subsections.

### 2.1. Model Interaction/Mixing

For given states $x_{k-1}^{j}=x_{k-1|k-1}^{j}$ with corresponding covariances $P_{k-1}^{j}=P_{k-1|k-1}^{j}$ and mixing probabilities $\mu_{k-1|k-1}^{i|j}$ for every model, the initial condition for the model *j* is:

(6) $${\hat{x}}_{k-1|k-1}^{0j}=\sum_{i=1}^{r}{\hat{x}}_{k-1|k-1}^{i}\mu_{k-1|k-1}^{i|j},\;\;j=1,2,...,r$$

The covariance corresponding to the above is:

(7) $$P_{k-1|k-1}^{0j}=\sum_{i=1}^{r}\mu_{k-1|k-1}^{i|j}\{P_{k-1|k-1}^{i}+[{\hat{x}}_{k-1|k-1}^{i}-{\hat{x}}_{k-1|k-1}^{0j}]{\left[{\hat{x}}_{k-1|k-1}^{i}-{\hat{x}}_{k-1|k-1}^{0j}\right]}^{T}\}$$

The model transition probabilities, which are related to Markov chain, are defined as:

(8) $$\pi_{\mathrm{ij}}=p\{M_{k}^{j}|M_{k-1}^{i}\}=\left[\begin{array}{cccc} \pi_{11} & \pi_{12} & \ldots & \pi_{1j}\\ \pi_{21} & \pi_{22} & \ldots & \pi_{2j}\\ \vdots & \vdots & \ddots & \vdots \\ \pi_{i1} & \pi_{i2} & \ldots & \pi_{\mathrm{ij}} \end{array}\right]$$

where *i*, *j* = 1,2,…,*r*, and *r* is the number of sub-models. Calculating the mixing probabilities with mode switching probability matrix *π_ij_* and the Gaussian mixing probabilities are computed via the equations:

(9) $$\mu_{k-1|k-1}^{i|j}=\frac{1}{{\bar{c}}_{j}}\pi_{\mathrm{ij}}\mu_{k-1}^{i}$$

where **c̄***_j_* is a normalization factor:

(10) $${\bar{c}}_{j}=\sum_{i=1}^{r}\pi_{\mathrm{ij}}\mu_{k-1}^{i}$$

### 2.2. Model Individual Filtering

- *Step 1 in UKF loop*. The unscented transform creates 2*n*+ 1 sigma vectors **X** (a capital letter) and weighted points *W*. For state estimation at instant *k* − 1, sigma points are generated according to:
  (11) $$\{\begin{array}{l} X_{0,k-1}^{j}={\hat{x}}_{k-1}^{0j}\\ X_{i,k-1}^{j}={\hat{x}}_{k-1}^{0j}+{\left(\sqrt{(n+\lambda )P_{k-1}^{0j}}\right)}_{i}^{T}\\ X_{i+n,k-1}^{j}={\hat{x}}_{k-1}^{0j}-{\left(\sqrt{(n+\lambda )P_{k-1}^{0j}}\right)}_{i}^{T} \end{array},i=1,...,n$$
  where ${\left(\sqrt{(n+\lambda )P_{k-1}^{0j}}\right)}_{i}$ is the *i* th row (or column) of the matrix square root. $\sqrt{(n+\lambda )P_{k-1}^{0j}}$ can be obtained from the lower-triangular matrix of the Cholesky factorization; *λ* = *α*^2^(*n* + *k*) − *n* is a scaling parameter used to control the covariance matrix; *α* determines the spread of the sigma points and is usually set to a small positive (e.g., 1*e* − 4 ≤ *α* ≤ 1); *k* is a secondly scaling parameter (usually set as 0); *β* is used to incorporate prior knowledge of the distribution of **x̄** (when **x** is normally distributed, *β* = 2 is an optimal value); $W_{i}^{\left(m\right)}$ is the weight for the mean associated with the *i* th point; and $W_{i}^{\left(c\right)}$ is the weight for the covariance associated with the *i* th point:
  (12a) $$W_{0}^{\left(m\right)}=\frac{\lambda }{\left(n+\lambda \right)}$$
  (12b) $$W_{0}^{\left(c\right)}=W_{0}^{\left(m\right)}+(1-\alpha^{2}+\beta )$$
  (12c) $$W_{i}^{\left(m\right)}=W_{i}^{\left(c\right)}=\frac{1}{2(n+\lambda )},\;\;i=1,...,2n$$
- *Step 2 in UKF loop.* Time update (prediction steps):
  (13) $$\zeta_{i,k|k-1}^{j}=f_{j}(X_{i,k|k-1}^{j}),\;\;\;i=0,...,2n$$
  (14) $${\hat{x}}_{k|k-1}^{j}=\sum_{i=0}^{2n}W_{i}^{\left(m\right)}\zeta_{i,k|k-1}^{j}$$
  (15) $$P_{k|k-1}^{j}=\sum_{i=0}^{2n}W_{i}^{\left(c\right)}[\zeta_{i,k|k-1}^{j}-{\hat{x}}_{k|k-1}^{j}]{\left[\zeta_{i,k|k-1}^{j}-{\hat{x}}_{k|k-1}^{j}\right]}^{T}+Q_{k-1}^{j}$$
  (16) $$Z_{i,k|k-1}^{j}=h(\zeta_{i,k|k-1}^{j})$$
  (17) $${\hat{z}}_{k|k-1}^{j}=\sum_{i=0}^{2n}W_{i}^{\left(m\right)}Z_{i,k|k-1}^{j}$$
- *Step 3 in UKF loop.* Measurement update (correction steps):
  (18) $$P_{\mathrm{zz}}^{j}=\sum_{i=0}^{2n}W_{i}^{\left(c\right)}[Z_{i,k|k-1}^{j}-{\hat{z}}_{k|k-1}^{j}]{\left[Z_{i,k|k-1}^{j}-{\hat{z}}_{k|k-1}^{j}\right]}^{T}+R_{k}^{j}$$
  (19) $$P_{\mathrm{xz}}^{j}=\sum_{i=0}^{2n}W_{i}^{\left(c\right)}[\zeta_{i,k|k-1}^{j}-{\hat{x}}_{k|k-1}^{j}]{\left[Z_{i,k|k-1}^{j}-{\hat{z}}_{k|k-1}^{j}\right]}^{T}$$
  (20) $$K_{k}^{j}=P_{\mathrm{xz}}^{j}{\left(P_{\mathrm{zz}}^{j}\right)}^{-1}$$
  (21) $${\hat{x}}_{k|k}^{j}={\hat{x}}_{k|k-1}^{j}+K_{k}^{j}\upsilon_{k}^{j},\;\;\text{where}\;\;\upsilon_{k}^{j}=z_{k}-{\hat{z}}_{k|k-1}^{j}$$
  (22) $$P_{k|k}^{j}=P_{k|k-1}^{j}-K_{k}^{j}P_{\mathrm{zz}}^{j}K_{k}^{j^{T}}$$

The samples are propagated through true nonlinear equations; the linearization is unnecessary (Calculation of the Jacobian matrix is not required).

### 2.3. Model Probabilities Update

The model probability $\mu_{k}^{j}$ is updated according to the model likelihood and model transition probability governed by the finite-state Markov chain:

(23) $$\mu_{k}^{j}=\frac{1}{c}\Lambda_{k}^{j}{\bar{c}}_{j}$$

where:

(24) $$c=\sum_{j=1}^{r}{\bar{c}}_{j}\Lambda_{k}^{j}$$

and $\Lambda_{k}^{j}$ is a likelihood function given by:

(25) $$\Lambda_{k}^{j}=\frac{1}{\sqrt{2\pi |P_{\mathrm{zz}}^{j}|}}\text{exp}\left[-\frac{1}{2}\upsilon_{k}^{j^{T}}{\left(P_{\mathrm{zz}}^{j}\right)}^{-1}\upsilon_{k}^{j}\right]$$

### 2.4. Combination of State Estimation and Covariance Combination

The model-individual estimates and covariances are combined to an overall state and covariance:

(26) $${\hat{x}}_{k|k}=\sum_{j=1}^{r}{\hat{x}}_{k|k}^{j}\mu_{k}^{j}$$

(27) $$P_{k|k}=\sum_{j=1}^{r}\mu_{k}^{j}\{P_{k|k}^{j}+[{\hat{x}}_{k|k}^{j}-{\hat{x}}_{k|k}]{\left[{\hat{x}}_{k|k}^{j}-{\hat{x}}_{k|k}\right]}^{T}\}$$

## 3. The Proposed Fuzzy Adaptive Filter Strategy

**Figure 1.**
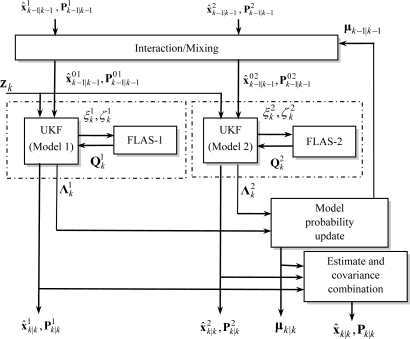
The block diagram of the FUZZY-IMMUKF algorithm. (one cycle with two models).

Figure 1 shows the block diagram for implementation of the proposed FUZZY-IMMUKF algorithm. The block on the right hand side, indicated by the dashed-line, is the fuzzy logic adaptive system (FLAS) for determining the value of process noise covariance. The rest represents the IMMUKF loop.

### 3.1. The Fuzzy Logic Adaptive System (FLAS)

**Figure 2.**
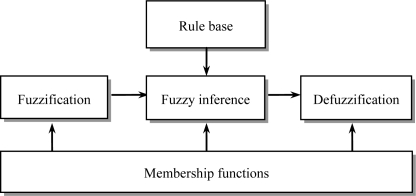
A fuzzy system.

Fuzzy logic was first developed by Zadeh in the mid-1960s for representing uncertain and imprecise knowledge. It provides an approximate but effective means of describing the behavior of systems that are too complex, ill-defined, or not easily analyzed mathematically. A typical fuzzy system consists of three components, that is, fuzzification, fuzzy reasoning (fuzzy inference), and fuzzy defuzzification, as shown in Figure 2. The fuzzification process converts a crisp input value to a fuzzy value, the fuzzy inference is responsible for drawing calculations from the knowledge base, and the fuzzy defuzzification process converts the fuzzy actions into a crisp action.

The fuzzification modules: (1) transforms the error signal into a normalized fuzzy subset consisting of a subset for the range of the input values and a normalized membership function describing the degree of confidence of the input belonging to this range; (2) selects reasonable and good, ideally optimal, membership functions under certain convenient criteria meaningful to the application. The characteristics of the fuzzy adaptive system depend on the fuzzy rules and the effectiveness of the rules directly influences its performance. To obtain the best deterministic output from a fuzzy output subset, a procedure for its interpretation, known as defuzzification should be considered. The defuzzification is used to provide the deterministic values of a membership function for the output. Using fuzzy logic to infer the consequent of a set of fuzzy production rules invariably leads to fuzzy output subsets.

The fuzzy modeling is the method of describing the characteristics of a system using fuzzy inference rules. In this paper, a Takagi-Sugeno (T-S) fuzzy system is used to detect the divergence of EKF and adapt the filter. Takagi and Sugeno proposed a fuzzy modeling approach to model nonlinear systems.

The output is the weighted average of the *y_k_*:

(28) $$y=\sum_{k=1}^{M}w_{k}.y_{k}$$

where the weights *w_k_* are computed as:

(29) $$w_{k}=\frac{\prod_{i=1}^{n}\mu_{F_{i}^{k}}(x_{i})}{\sum_{j=1}^{M}\left[\prod_{i=1}^{n}\mu_{F_{i}^{j}}(x_{i})\right]}$$

with $\sum_{i=1}^{M}w_{i}=1$, and the *μ*’s represent the membership function.

### 3.2. The Fuzzy Adaptive System Based on Unscented Kalman Filter

The degree of divergence (DOD) parameters for identifying the degree of change in vehicle dynamics needs to be defined. Examples for possible approaches are given as follows. The innovation information at the present epoch is employed for timely reflect the change in vehicle dynamics. The DOD parameter *ξ* can be defined as the trace of innovation covariance matrix at present epoch (*i.e*., the window size is one) divided by the number of satellites employed for navigation processing:

(30) $$\xi =\frac{1}{m}\sum_{i=1}^{m}\upsilon_{i}\upsilon_{i}^{T}$$

where **υ***_k_* = [*υ*_1_ *υ*_2_…*υ_m_*]*^T^*, *m* is the number of measurements (number of satellites). Furthermore, the averaged magnitude of innovation at the present epoch can also be used:

(31) $$\zeta =\frac{1}{m}\sum_{i=1}^{m}\left|\upsilon_{i}\right|$$

**Figure 3.**
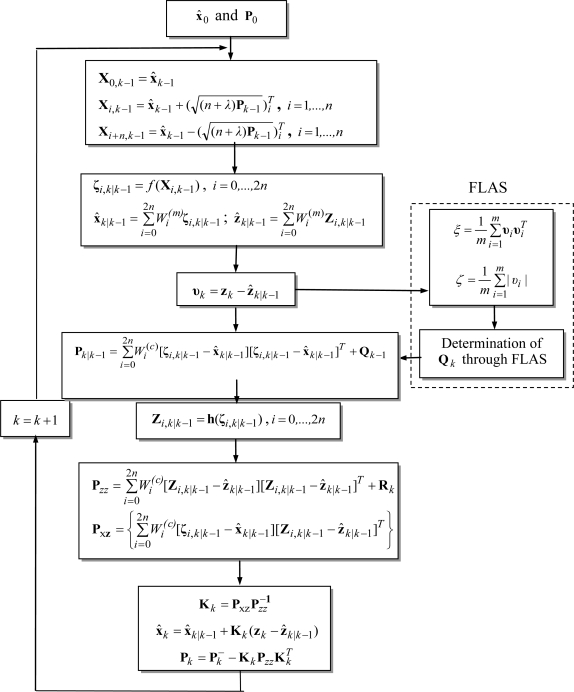
The flow chart of the FLAS-UKF.

In the FLAS, the DOD parameters are employed as the inputs for the fuzzy inference engines. By monitoring the DOD parameters, the FLAS is able to on-line determine better lower and upper bounds of the process noise covariance according to the innovation information, and therefore improves the estimation performance. The flow chart of the FLAS-UKF algorithm is shown in Figure 3.

## 4. Navigation Integration Processing and Performance Evaluation

**Figure 4.**
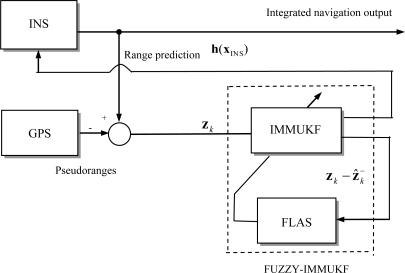
Configuration of the tightly-coupled feedback integrated navigation using the proposed approach.

Simulation experiments have been carried out to evaluate the performance of the proposed approach in comparison with the conventional methods for GPS/INS integrated navigation system processing. The tightly-coupled configuration is employed for demonstration. The commercial software Satellite Navigation (SATNAV) Toolbox by GPSoft LLC was employed for generating the satellite positions and pseudoranges. The satellite constellation was simulated and the error sources corrupting GPS measurements include ionospheric delay, tropospheric delay, receiver noise and multipath. Assume that the differential GPS (DGPS) mode is used and most of the errors can be corrected, but the multipath and receiver thermal noise cannot be eliminated. Figure 4 shows the configuration of the tightly-coupled feedback GPS/INS integrated navigation processing based on the FLAS-coupled IMMUKF filtering mechanism. The measurement is the residual between GPS pseudorange and INS predicted range, which is used as the measurement of the UKF.

**Figure 5.**
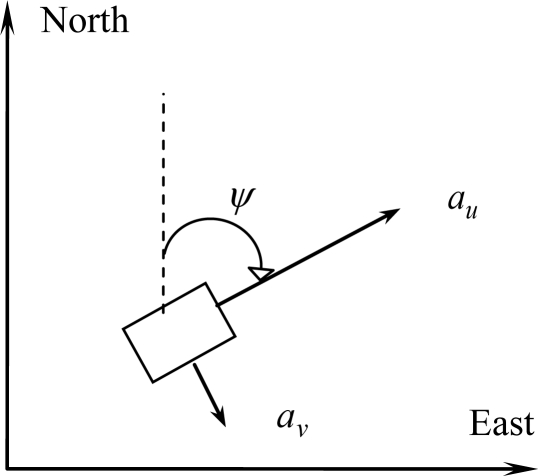
Illustration of the two-dimensional inertial navigation.

The differential equations describing the two-dimensional inertial navigation state are:

(32) $$\left[\begin{array}{c} \dot{n}\\ \dot{e}\\ {\dot{v}}_{n}\\ {\dot{v}}_{e}\\ \dot{\psi } \end{array}\right]=\left[\begin{array}{c} v_{n}\\ v_{e}\\ a_{n}\\ a_{e}\\ \omega_{r} \end{array}\right]=\left[\begin{array}{c} v_{n}\\ v_{e}\\ \text{cos}(\psi )a_{u}-\text{sin}(\psi )a_{v}\\ \text{sin}(\psi )a_{u}+\text{cos}(\psi )a_{v}\\ \omega_{r} \end{array}\right]$$

where [*a_u_*, *a_v_*] are the measured acceleration in the body frame, *ω_r_* is the measured yaw rate in the body frame, as shown in Figure 5. The error model for INS is augmented by some sensor error states such as accelerometer biases and gyroscope drifts. Actually, there are several random errors associated with each inertial sensor. It is usually difficult to set a certain stochastic model for each inertial sensor that works efficiently at all environments and reflects the long-term behavior of sensor errors. The difficulty of modeling the errors of INS raised the need for a model-less GPS/INS integration technique. Linearization of Equation (32) results in the following set of linearized equations:

(33) $$\left[\begin{array}{c} \delta \dot{n}\\ \delta \dot{e}\\ \delta {\dot{v}}_{n}\\ \delta {\dot{v}}_{e}\\ \delta \dot{\psi } \end{array}\right]=\left[\begin{array}{ccccc} 0 & 0 & 1 & 0 & 0\\ 0 & 0 & 0 & 1 & 0\\ 0 & 0 & 0 & 0 & 0\\ 0 & 0 & 0 & 0 & 0\\ 0 & 0 & 0 & 0 & 0 \end{array}\right]\left[\begin{array}{c} \delta n\\ \delta e\\ {\delta v}_{n}\\ {\delta v}_{e}\\ \delta \psi \end{array}\right]+\left[\begin{array}{c} 0\\ 0\\ w_{n}\\ w_{e}\\ w_{\psi } \end{array}\right]$$

which will be utilized in the integration Kalman filter as the inertial error model. In Equation (33), *δn* and *δe* represent the east, and north position errors; *δv_n_* and *δv_e_* represent the east, and north velocity errors; and *δψ* represent yaw angle.

The measurement model is written as:

(34) $$\left[\begin{array}{c} \rho_{1}\\ \rho_{2}\\ \vdots \\ \rho_{n} \end{array}\right]=\left[\begin{array}{c} {\hat{\rho }}_{1}\\ {\hat{\rho }}_{2}\\ \vdots \\ {\hat{\rho }}_{n} \end{array}\right]+\left[\begin{array}{ccccc} h_{11} & h_{12} & 0 & 0 & 0\\ h_{21} & h_{22} & 0 & 0 & 0\\ \vdots & \vdots & \vdots & \vdots & \vdots \\ h_{n1} & h_{n2} & 0 & 0 & 0 \end{array}\right]\left[\begin{array}{c} \delta n\\ \delta e\\ {\delta v}_{n}\\ {\delta v}_{e}\\ \delta \psi \end{array}\right]+\left[\begin{array}{c} v_{1}\\ v_{2}\\ \vdots \\ v_{n} \end{array}\right]$$

**Figure 6.**
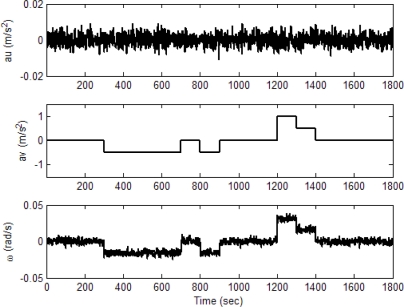
Simulated sensor outputs for the accelerometers and gyroscope.

**Figure 7.**
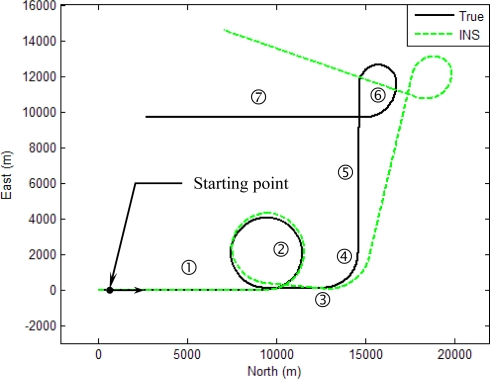
Trajectories for the simulated vehicle (solid) and the INS derived position (dashed).

**Figure 8.**
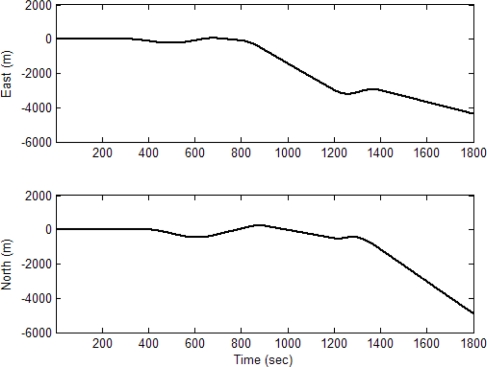
East and north components of INS navigation errors.

The experiment was conducted on a simulated vehicle trajectory originating from the (0,0,0) m location in the ENU coordinate frame. The simulated sensor outputs for the accelerometers and gyroscope are shown as in Figure 6. The schematic illustration of trajectories for the simulated vehicle and the unaided INS derived position is shown in Figure 7. The trajectory can be divided mainly into seven time intervals (or segments), as indicated on the figure, according the dynamic characteristics. Further detailed description of the vehicle motion is given in Table 1. The vehicle was simulated to conduct constant-velocity straight-line during the four time intervals, 0–300, 701–800, 901–1,200 and 1,401–1,800 s, all at a speed of 10*π* m/s. Furthermore, it conducted circular motion with radius 2,000 meters during 301–700 s (counterclockwise) and 801–900 s (counterclockwise). The vehicle conducted the third circular motion with radius 1,000 meters (clockwise) during 1,201–1,400 s, where medium and high dynamic maneuverings are involved. The following parameters were used: the values of noise standard deviation are 3e-3 m/s^2^ for accelerometers and gyroscopes. Figure 8 shows the trajectories for the simulated vehicle and the INS derived position, where the error grows unbounded. Assume that the differential GPS (DGPS) mode is used and most of the errors can be corrected, but the multipath and receiver thermal noise cannot be eliminated. The measurement noise variances *r_ρi_* are assumed *a priori* known, which is set as 4 *m*^2^. The measurement noise covariance matrix is given by R*_k_* = 100 × *I*_*m* × *m*_.

The following model transition probability matrices of the Markov chain *π_ij_* were assumed:

(35) $$\pi_{\mathrm{ij}}=\{\begin{array}{cc} 0.99 & \text{if}\;i=j\\ \frac{1-0.99}{N-1} & \mathrm{otherwise} \end{array}$$

In this paper, two models are employed, therefore *N* = 2 and:

(36) $$\pi_{\mathrm{ij}}=\left[\begin{array}{cc} 0.99 & 0.01\\ 0.01 & 0.99 \end{array}\right]$$

The initial model probability for each sub-model is chosen as:

(37) $$\mu_{0}^{j}=\{\begin{array}{cc} 0.5 & \text{if}\;j=1\\ \frac{1-0.5}{N-1} & \mathrm{otherwise} \end{array}$$

The process noise covariance matrix is given by:

(38) $$Q_{k}=\left[\begin{array}{ccccc} 0 & 0 & 0 & 0 & 0\\ 0 & {4e}^{-4} & 0 & 0 & 0\\ 0 & 0 & 0 & 0 & 0\\ 0 & 0 & 0 & {4e}^{-4} & 0\\ 0 & 0 & 0 & 0 & {4e}^{-5} \end{array}\right]$$

and the parameters utilized in the UKF are given as follows: *α* = 1*e* − 4, *β* = 2 and *k* = 0. In addition, the parameters utilized in the STA of the STUKF are: *η* = 0.6, *ρ* = 0.1, and the softening factor *ɛ* = 4.5. The sigma points capture the same mean and covariance irrespective of the choice of matrix square root which is used. The numerical efficient and stable method such as the Cholesky factorization has been used in obtaining the sigma points.

**Figure 9.**
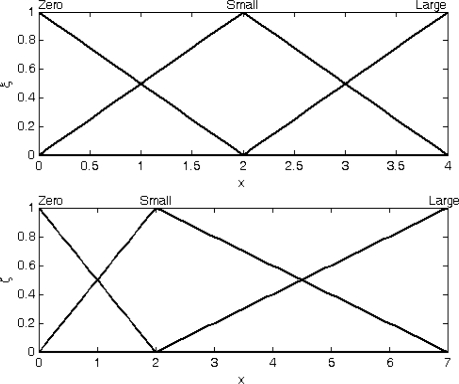
Membership functions of input fuzzy variables *ξ* (top) and *ζ* (bottom).

The membership functions (MFs) of input fuzzy variable DOD parameters as shown in Figure 9 are triangle MFs. The presented FLAS is the *If-Then* form and consists of nine rules:

1. IF *ξ* is zero and *ζ* is zero THEN **Q***_k_* is 1
2. IF *ξ* is zero and *ζ* is small THEN **Q***_k_* is 1
3. IF *ξ* is zero and *ζ* is large THEN **Q***_k_* is 1
4. IF *ξ* is small and *ζ* is zero THEN **Q***_k_* is 1
5. IF *ξ* is small and *ζ* is small THEN **Q***_k_* is *ξ* + *ζ* + 4
6. IF *ξ* is small and *ζ* is large THEN **Q***_k_* is *ξ* + *ζ* + 4
7. IF *ξ* is large and *ζ* is zero THEN **Q***_k_* is 6*ξ* + 6*ζ* + 12
8. IF *ξ* is large and *ζ* is small THEN **Q***_k_* is 6*ξ* + 6*ζ* + 12
9. IF *ξ* is large and *ζ* is large THEN **Q***_k_* is 6*ξ* + 6*ζ* + 12

**Figure 10.**
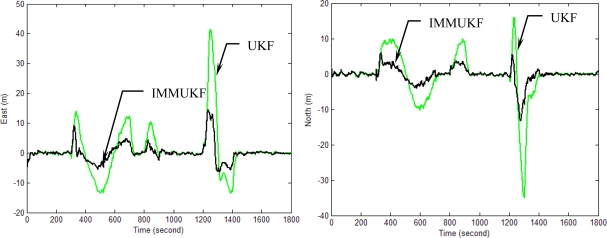
Comparison of the navigation accuracy for UKF and IMMUKF.

**Figure 11.**
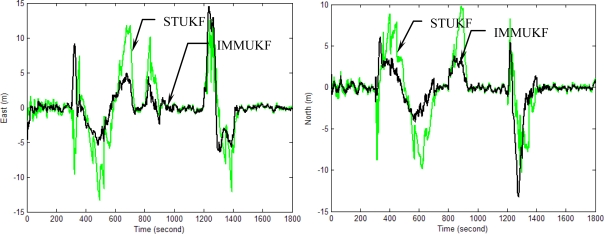
Comparison of the navigation accuracy for IMMUKF and STUKF.

**Figure 12.**
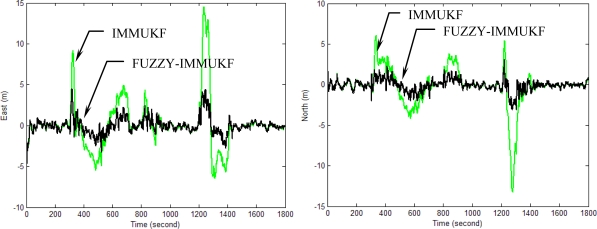
Comparison of the navigation accuracy for IMMUKF and FUZZY-IMMUKF.

**Figure 13.**
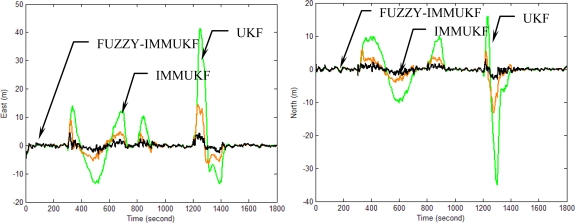
Comparison of the navigation accuracy for three approaches: FUZZY-IMMUKF, IMMUKF and UKF.

**Figure 14.**
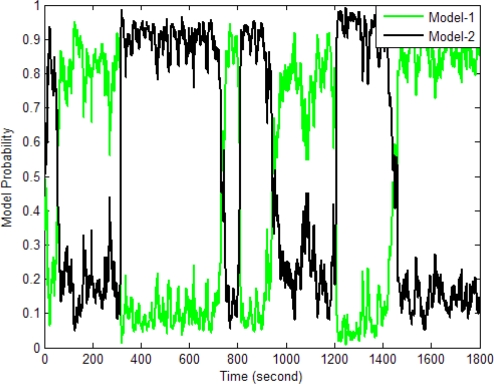
Model probability of FUZZY-IMMUKF.

**Figure 15.**
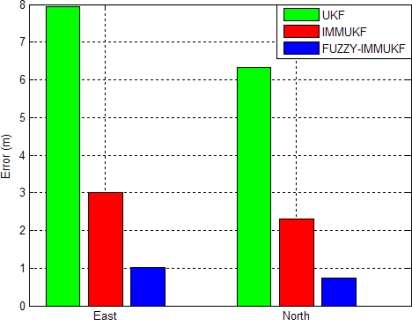
Comparisons of east and north position RMS errors via three approaches: UKF, IMMUKF and FUZZY-IMMUKF.

Figures 10–13 provide the navigation results for the UKF, IMMUKF, STUKF and FUZZY-IMMUKF approaches. Before the FLAS was incorporated, preliminary evaluation on the adaptation algorithms were carried out. Comparisons of navigation accuracies for the standard UKF and IMMUKF, and for the IMMUKF and STUKF, are shown in Figures 10 and 11, respectively. Both the IMM and STA demonstrate the adaptation capability. Comparison of IMMUKF and STUKF in Figures 11 is included for better understanding the performance of the two algorithms, *i.e.*, parametric adaptation and structural adaptation. The proposed FUZZY-IMMUKF has two main features. First, it employs the IMM mechanism for adjusting the adequate model based on the dynamic characteristics. Furthermore, the FLAS is adopted for automatically determining the lower and upper bounds of **Q***_k_*. In is seen that substantial estimation accuracy improvement can be obtained by using the proposed method. In addition, to inspect the correctness of the filter switching capability, the mode probability of the FUZZY-IMMUKF needs to be checked, which is depicted in Figure 14. The soft-switching capability enables the filter to capture the adequate vehicle dynamic. Figure 15 provides comparison of east and north position root mean squared errors via the three approaches: UKF, IMMUKF and FUZZY-IMMUKF. Table 2 shows the comparison of navigation RMS (root mean square) errors for the three approaches.

Some remarks can be made as follows:

1. In the four time intervals, 0–300, 701–800, 901–1,200 and 1,401–1,800 s, the vehicle is not maneuvering and is conducting constant-velocity straight-line motion. For this case, all the UKF, IMMUKF and FUZZY-IMMUKF provide equivalently good results. The navigation accuracies based on the three approaches have relatively small difference. By use of the T-S fuzzy logic, the FLAS senses smaller values of DOD parameters, and then reduces the process noise covariance. As a result, the navigation accuracies based on the UKF, IMMUKF and FUZZY-IMMUKF are equivalent.
2. In the three time intervals, 301–700, 801–900 and 1,201–1,400 s, the vehicle is maneuvering. The mismatch of the model leads to larger navigation error while the FLAS timely detects the increase of DOD parameter, and determines a large process noise covariance to maintain good estimation accuracy. It has been verified that, by monitoring the innovation information, the FUZZY-IMMUKF has good capability to detect the change in vehicle dynamics and adjust the process noise covariance for preventing the divergence and achieving better navigation accuracy.

## 5. Conclusions

The classical unscented Kalman filter does not possess the capability to monitor parameter changes due to changes in vehicle dynamics. An interacting multiple-model based method is suggested to improve the unscented Kalman filter for better navigation data fusion. The resulting IMMUKF ensures better description on the vehicle dynamics and exhibits superior navigation accuracy when compared with the classical UKF algorithm. This paper has presented a fuzzy interacting multiple model unscented Kalman filter for GPS/INS navigation processing to prevent the divergence problem in high dynamic environments.

The fuzzy system is employed for dynamically adjusting the lower and upper bounds of the process noise covariance, which will be used in each of the parallel filters in the IMM architecture by monitoring the innovation information so as to provide further improvement in estimation accuracy. Through the proposed approach, lower order of filter model can be utilized and, therefore, less computational effort will be sufficient without compromising estimation accuracy significantly. When a designer does not have sufficient information to develop the complete filter models or when the filter parameters are slowly changing with time, the fuzzy system can be employed to enhance the IMMUKF performance. These performance comparisons of UKF, IMMUKF and FUZZY-IMMUKF have been conducted and the proposed FUZZY-IMMUKF algorithm has demonstrated very promising results in navigational accuracy. Future research can be conducted to undertake the implementation of the following issues.

The current work essentially shows the feasibility of the approach. Evaluation of the proposed approach on a real system with better design of the fuzzy logic might be considered in the near future. Other artificial intelligence such as neural work may also be incorporated for better tuning. Furthermore, the GPS RAIM (Receiver Autonomous Integrity Monitoring) in civil aviation might also be considered as one of the potential applications.

## Figures and Tables

**Table 1.** Description of the vehicle motion.

**Segment number**	**Time interval (sec)**	**Motion**
1	[0–300]	Constant velocity straight line
2	[301–700]	Circular motion (counter-clockwise)
3	[701–800]	Constant velocity straight line
4	[801–900]	Counter-clockwise turn
5	[901–1,200]	Constant velocity straight line
6	[1,201–1,400]	Clockwise turn
7	[1,401–1,800]	Constant velocity straight line

**Table 2.** Position accuracies for the three approaches (RMS errors, in meters).

	
	**East**	**North**
**UKF**	7.9530	6.3195
**IMMUKF**	2.9967	2.3021
**FUZZY-IMMUKF**	1.0184	0.7286

## Appendix

### A. The Unscented Kalman Filter

Consider an *n* dimensional random variable **x**, having the mean **x̂** and covariance **P**, and suppose that it propagates through an arbitrary nonlinear function *f*. The unscented transform creates 2*n* + 1 sigma vectors **X** (a capital letter) and weighted points *W*, given by:

(39) $$\begin{array}{c} X_{\left(0\right)}=\hat{x}\\ X_{\left(i\right)}=\hat{x}+{\left(\sqrt{(n+\lambda )P}\right)}_{i}^{T},\;\;i=1,...,n\\ X_{\left(i+n\right)}=\hat{x}-{\left(\sqrt{(n+\lambda )P}\right)}_{i}^{T},\;\;i=1,...,n \end{array}$$

$W_{i}^{\left(m\right)}$ and $W_{i}^{\left(c\right)}$ are the weighs for the mean and covariance, respectively, associated with the i *th* point:

(40a) $$W_{0}^{\left(m\right)}=\frac{\lambda }{\left(n+\lambda \right)}$$

(40b) $$W_{0}^{\left(c\right)}=W_{0}^{\left(m\right)}+(1-\alpha^{2}+\beta )$$

(40c) $$W_{i}^{\left(m\right)}=W_{i}^{\left(c\right)}=\frac{1}{2(n+\lambda )},\;\;i=1,...,2n$$

The sigma vectors are propagated through the nonlinear function to yield a set of transformed sigma points, **y***_i_* = *f*(**X***_i_*), *i* = 0,…, 2*n*.

The implementation algorithm of UKF is summarized as follows:

1. The transformed set is given by instantiating each point through the process model:
  (41) $$\zeta_{i,k|k-1}=f(X_{i,k|k-1}),\;\;i=0,...,2n$$
2. Predicted mean:
  $${\hat{x}}_{k|k-1}=\sum_{i=0}^{2n}W_{i}^{\left(m\right)}\zeta_{i,k|k-1}$$
3. Predicted covariance:
  (42) $$P_{k|k-1}=\sum_{i=0}^{2n}W_{i}^{\left(c\right)}[\zeta_{i,k|k-1}-{\hat{x}}_{k|k-1}]{\left[\zeta_{i,k|k-1}-{\hat{x}}_{k|k-1}\right]}^{T}+Q_{k-1}$$
4. Instantiate each of the prediction points through observation model:
  $$Z_{i,k|k-1}=h(\zeta_{i,k|k-1})$$
5. Predicted observation:
  $${\hat{z}}_{k|k-1}=\sum_{i=0}^{2n}W_{i}^{\left(m\right)}Z_{i,k|k-1}$$
6. Innovation covariance:
  (43) $$P_{\mathrm{zz}}=\sum_{i=0}^{2n}W_{i}^{\left(c\right)}[Z_{i,k|k-1}-{\hat{z}}_{k|k-1}]{\left[Z_{i,k|k-1}-{\hat{z}}_{k|k-1}\right]}^{T}+R_{k}$$
7. Cross covariance:
  (44) $$P_{\text{xz}}=\sum_{i=0}^{2n}W_{i}^{\left(c\right)}[\zeta_{i,k|k-1}-{\hat{x}}_{k|k-1}]{\left[Z_{i,k|k-1}-{\hat{z}}_{k|k-1}\right]}^{T}$$
8. Performing update:
  (45) $$\begin{array}{c} K_{k}=P_{\text{xz}}P_{\mathrm{zz}}^{-1}\\ {\hat{x}}_{k}={\hat{x}}_{k|k-1}+K_{k}(z_{k}-{\hat{z}}_{k|k-1})\\ P_{k}=P_{k}^{-}-K_{k}P_{\mathrm{zz}}K_{k}^{T} \end{array}$$

### B. The Strong Tracking Algorithm

Based on the idea as in the AKF, the synthesis of UKF and strong tracking filter leads to the strong tracking unscented Kalman filter (STUKF). The suboptimal fading factors are introduced into the nonlinear smoother algorithm:

(46) $$s_{i,k}=\frac{\mathrm{tr}[{\eta V}_{k}-{ɛR}_{k}]}{\mathrm{tr}[P_{\mathrm{zz}}]}=\{\begin{array}{ll} s_{i,k}, & s_{i,k}>1\\ 1, & s_{i,k}\leq 1 \end{array}$$

(47) $$V_{k}=\{\begin{array}{l} \upsilon_{0}\upsilon_{0}^{T}\\ \frac{\left[\rho V_{k-1}+\upsilon_{k}\upsilon_{k}^{T}\right]}{1+\rho },\;\;\;\;\;\;k\geq 2 \end{array}$$

The covariance matrix needs to be updated the following way. The new $P_{k}^{-}$ needs to be modified and can be obtained by multiplying Equation (42) by the factor **S***_k_*:

(48) $$P_{k|k-1}=S_{k}\left\{\sum_{i=0}^{2n}W_{i}^{\left(c\right)}[\zeta_{i,k|k-1}-{\hat{x}}_{k|k-1}]{\left[\zeta_{i,k|k-1}-{\hat{x}}_{k|k-1}\right]}^{T}+Q_{k}\right\}$$

Similarly, the covariance matrix **P***_zz_* and **P***_xz_*, as represented by Equations (43) and (44), also respectively, can be modified and rewritten as:

(49) $$P_{\mathrm{zz}}=S_{k}\left\{\sum_{i=0}^{2n}W_{i}^{\left(c\right)}[Z_{i,k|k-1}-{\hat{z}}_{k|k-1}]{\left[Z_{i,k|k-1}-{\hat{z}}_{k|k-1}\right]}^{T}+R_{k}\right\}$$

(50) $$P_{\text{xz}}=S_{k}\left\{\sum_{i=0}^{2n}W_{i}^{\left(c\right)}[\zeta_{i,k|k-1}-{\hat{x}}_{k|k-1}]{\left[Z_{i,k|k-1}-{\hat{z}}_{k|k-1}\right]}^{T}\right\}$$

where **S***_k_* = *diag* (*s*_1_, *s*_2_ …,*s_m_*). One approach is to assign the scale factors as constants. When *s_i_* ≤ 1 (*i* = 1,2,…,*m*), the filtering is in a steady state processing while *s_i_* > 1, the filtering may tend to be unstable. For the case *s_i_* = 1, it deteriorates to the standard Kalman filter. Further detailed information for the strong tracking unscented Kalman filter can be referred to Reference [9].
